# Does visual attention to diagnostic facial features predict eyewitness identification accuracy? Implications for diagnostic feature detection theory

**DOI:** 10.1371/journal.pone.0355167

**Published:** 2026-09-22

**Authors:** Pia Pennekamp, Michelle M. Ramey, Dilhan Töredi, James Michael Lampinen

**Affiliations:** 1 Department of Psychological Science, University of Arkansas, Fayetteville, Arkansas, United States of America; 2 Department of Psychology, Åbo Akademi University, Turku, Finland; Julius-Maximilians-Universität Würzburg: Julius-Maximilians-Universitat Wurzburg, GERMANY

## Abstract

A police lineup includes a suspect and known-innocent fillers. The purpose of fillers is to protect an innocent suspect as witnesses must rely on their memory to make a lineup decision (i.e., select or reject). Diagnostic Feature Detection theory proposes that witnesses compare facial features across lineup members, weighting shared and non-shared features differently during the decision process (Wixted & Mickes, 2014). According to this view, only non-shared features are diagnostic because they provide distinguishing information, compared to features that are shared amongst all lineup members. Using eyetracking, we examined eyewitnesses’ viewing behavior and decision making in lineups when features amongst lineup members were shared. After encoding twelve target faces (six Black, six White), participants (*n* = 100) made identification decisions on target-present lineups (one lineup per target), with lineup members presented either simultaneously or sequentially, providing confidence after each lineup decision. In the shared-feature lineups, we digitally edited one of the target’s features (nose, eyes, or mouth) onto all lineup members so that they shared that feature (e.g., in one lineup, all members had the target’s eyes). In the control lineups, we edited each lineup member’s own feature (nose, eyes, or mouth) to control for effects of photoshopping. Results indicate there were no differences in allocation of overt visual attention (via eye movements) to diagnostic features between lineup types or by accuracy. These findings do not support the proposition that eyewitnesses overtly evaluate shared features in lineups, or account for their diagnosticity via allocation of overt visual attention.

## Introduction

A lineup is an identification procedure in which a suspect is presented amongst a series of fillers and the witness is asked if any of the individuals is the guilty party. A suspect is a person who the police think might be guilty. If the suspect is guilty, the lineup is called a target present lineup. If the suspect is innocent, the lineup is called a target absent lineup. Fillers are known innocent individuals who ideally provide plausible alternatives to the suspect. If a filler is chosen by the eyewitness, it is an error. However, such errors are relatively harmless because the filler is known by police to be innocent. If a filler is chosen, the filler is not in jeopardy of being arrested. If the suspect is chosen, on the other hand, the suspect is in jeopardy of being arrested, prosecuted and imprisoned. Thus, an eyewitness choosing an innocent suspect is a dangerous error. The police already have their suspicions that the person is guilty, and the identification of the suspect by the witness further implicates the innocent person in the crime.

For this reason, a great deal of work has focused on developing best practices that minimize the chance that an innocent suspect will be chosen. These best practices have been formalized in recommendations by major scientific and law enforcement organizations [1–4]. One of the most important recommendations is that the fillers chosen for a lineup should match the general description of the witness as well as the police suspect does. Lineups in which this precondition is met are generally called *fair* lineups and lineups in which the suspect stands out as being distinctive are called *biased* lineups (or *unfair* lineups). Unfair lineups harm eyewitness accuracy (e.g., [5]). When a suspect stands out, eyewitnesses are more likely to make erroneous identifications of that suspect (e.g., [6,7]). Fair lineups, on the other hand, reduce innocent-suspect misidentifications and are thus a superior test of eyewitnesses’ memory for the perpetrator [6,8,9].

There has been substantial research examining the optimal level of suspect–filler similarity needed to maximize discriminability while preserving lineup fairness (e.g., [6]). For example, the most recent whitepaper suggests that systematically manipulating filler similarity to the lineup suspect, beyond simply ensuring that lineup members match the culprit’s description to preserve fairness, may offer advantages over traditional filler selection strategies, while acknowledging that the optimal level of suspect–filler similarity remains an open empirical question [4]. Converging evidence shows that, from fair lineups where all lineup members match the description, selecting fillers that are dissimilar to the suspect (i.e., low-similarity lineups) outperforms selecting fillers that are highly similar to the suspect (e.g., [10–12]; but see [13]). Low-similarity lineups enhance discriminability because they increase guilty-suspect identification rates from target-present lineups to a greater extent than they increase innocent-suspect identification rates from target-absent lineups [11,13,14]). This pattern of lineup decisions from low-similarity lineups are explained by a theory called the *Diagnostic Feature-Detection Theory*, or DFD [15].

According to DFD, identification processes from lineups involve comparing features of the lineup members to features of the witness’s memory representation of the perpetrator. Lineups which allow for these comparisons are thought to increase the ability of eyewitnesses to discriminate between guilty and innocent suspects (e.g., [15,16]). Because fillers are often selected based on their match to either the suspect’s appearance (i.e., match to suspect) or the witness’s description (i.e., match to description), or both (e.g., [6,10]), across lineup members there will be some features that are shared and some features that are unique to individual lineup members. Features that are shared among many lineup members are thought to have low informational value compared to features that are unique to individual lineup members. To that extent, DFD theorizes that eyewitnesses can distinguish between facial features that are diagnostic (i.e., unique to particular lineup members) versus non-diagnostic (i.e., shared amongst lineup members) and use this information to make lineup decisions. For example, if all lineup members have the same facial tattoo, this feature would be non-diagnostic; in contrast, if only one lineup member has a facial tattoo, this feature would be diagnostic.

DFD claims that viewing a suspect amongst a set of fillers allows the witness to optimally give more weight to diagnostic features and less weight to non-diagnostic features and that doing so increases their ability to discriminate innocent from guilty suspects [15,16]. There is research that suggests a DFD-like process does seem to occur. For example, Colloff et al. [17] tested performance of fair and unfair lineups using a distinctive feature (i.e., bruise). Colloff et al. suggest that unfair lineups impair witnesses’ ability to accurately distinguish between guilty and innocent suspects. In an unfair lineup, witnesses failed to discount a non-diagnostic feature but could appropriately discount it in a fair lineup when the suspect did not stand out; it was proposed that the fair lineup “gave more weight” to other, more informative cues ([17]; p. 1237). That is, similar-looking fillers in fair lineups allow shared facial features that are non-diagnostic of guilt to be noticed and discounted [17]. Carlson and colleagues (2019) [18] artificially generated lineup fillers to manipulate differences in one, two, or three facial features (eyes, nose, or mouth). As the number of varying features increased (thereby reducing the overall similarity among lineup members and increasing potentially diagnostic information), discriminability improved. These findings provide empirical support for the DFD framework.

Although these findings support DFD, the mechanisms underlying these effects are not yet known. In particular, DFD suggests that it is the evaluation of diagnosticity of features that enhances discriminability in fair lineups. Therefore, for discriminability to be enhanced, witnesses should spend less time assessing non-diagnostic features as they would appreciate these to be non-diagnostic. However, *how* eyewitnesses might assess and leverage diagnosticity of such features remains to be explained. One possible mechanism underlying DFD is that when people notice one or more features to be non-diagnostic, they may direct more overt visual attention to the features that are diagnostic. This view would predict that:

1) people should allocate more attention to diagnostic than non-diagnostic features, and
2) to the extent that people devote more attention to diagnostic features, they should be more accurate.

Indeed, note that Shen et al. [11] claim that “...witnesses presented with a simultaneous lineup immediately realize that certain features... do not vary across the lineup members...” (p. 454). A plausible implication of this claim is that witnesses quickly notice shared features and then they devote relatively little attention to them afterwards. Yet, these predictions have not been experimentally tested.

There has been considerable speculation about what processes might underlie DFD. It is unclear whether the evaluation of diagnostic versus non-diagnostic features is an aspect of decision making—and therefore might involve changes in the allocation of visual attention—or whether it might be an aspect of memory processing that occurs before or separately from decision making. Perhaps the best way to assess overt attention to different facial features is to track witnesses’ eyes while they make lineup decisions. Previous studies have assessed witnesses’ gaze behavior on a lineup task. For example, Flowe [19] reported that participants spent more time evaluating the suspect’s face in sequential lineups than simultaneous lineups but there were no differences in performance. Additionally, consistent with the possibility that witnesses may direct more attention to diagnostic features, evidence from the broader visual attention literature (e.g., using scenes) suggests that eye movements are directed toward stimulus features that have greater informational content. For example, gaze is more often directed to scene regions that contain more local semantic content or are more task-informative (e.g., [20–22]) and saccades are shorter in latency and duration when viewing a stimulus that is relevant to a task [23–26]. Eye movements thus provide a means to assess the extent to which people allocate attention to diagnostic features in a recognition task.

## Present study

In the present study, we tested whether allocating more overt attention (i.e., eye movements) to diagnostic relative to non-diagnostic features predicts better identification accuracy. To do this, we manipulated shared features in lineup members. That is, some of our lineups included features that were shared between all lineup members (i.e., non-diagnostic features). Although prior work suggests diagnostic features can improve discriminability (e.g., [18]), an important mechanism remains unexamined: whether participants direct greater visual attention to unshared features among lineup members. Eye tracking can index the extent to which people preferentially overtly attend to (or avoid) shared features compared to non-shared features. We therefore sought to examine eyewitnesses’ viewing behavior and decision-making in lineups when features amongst lineup members are shared (i.e., non-diagnostic) versus non-shared (i.e., diagnostic).

To improve ecological validity and applicability to a variety of lineup tasks, we also examined the extent to which effects varied between simultaneous and sequential lineups, and Black and White faces. Specifically, because a large body of literature has focused on the effect of lineup types on eyewitness discriminability [15,27–31], we sought to examine the effect of shared features in simultaneous and sequential lineups. In a simultaneous lineup, the suspect and fillers are presented at the same time which allows witnesses to compare and contrast lineup members. Sequential lineups, on the other hand, involve presenting one lineup member at a time. Witnesses examine each lineup member individually, which presumably would make it more difficult to determine which features are diagnostic versus non-diagnostic. We are not aware of any research that has employed eye tracking to investigate gaze behavior for Black and White faces in the context of lineups. Because some research suggests people differentially view faces of different races (see [32–34]), we sought to examine potential effects of the race of lineup members to improve ecological validity. We note that these considerations were secondary to our central objective—namely, investigating the extent to which eyewitnesses allocate overt attention to diagnostic features.

### Theoretical predictions

DFD proposes that discriminability is improved by fair lineups because eyewitnesses weigh non-shared features more heavily than shared features when evaluating lineup members [15,17,35]. One interpretation of this is that non-shared features should draw more visual attention than shared features, and that more attention to non-shared features should predict better accuracy. We thus expected that more time looking at non-shared facial features (i.e., more fixations, longer duration of fixations) relative to shared facial features would predict higher accuracy in identifying previously seen faces. We also expected that there may be temporal nuances to this effect: In particular, the above effect may occur after an initial assessment period in which eyewitnesses first determined which features were shared versus non-shared, before focusing on the non-shared features.

## Method

The study was approved by the university’s research ethics board. Informed consent was obtained electronically prior to commencement of the study. Data collection began October 3, 2023, and ended on April 12, 2024.

### Participants

We conducted an a priori power analysis based on Flowe [19], given that they used eye tracking to examine differences between simultaneous and sequential lineups using the same number of trials as the present experiment. A power analysis of the smallest relevant effect observed in Flowe [19]—viewing duration varying by lineup type between sequential and simultaneous lineups, η^2^ = .28—indicated that we would need to recruit 50 participants to achieve 99% power. However, because we planned to conduct temporal analyses within trials as well, we doubled our target sample size. Specifically, we recruited participants until we reached a sample of 100 participants with data that met our preselected criteria below. A sensitivity analysis suggested 80% power to detect a moderate effect, η^2^ = .075.

Participants (*N* = 164) were recruited via the university’s participant pool. Participants were required to have normal or corrected-to-normal vision. Based on our pre-registered exclusion criteria, we excluded data from participants with less than 75% eye tracking signal (e.g., more than 25% of the eye tracking samples were blinks), calibration worse than a degree error of.55 average and 1.05 max, or below-chance performance on memory judgments (*n* = 64 across all criteria). The usable sample of participants (*n* = 100) identified as female (68%), male (31%), and non-binary (1%). Participants self-identified as White (88%), Asian (5%), Black (2%), Hispanic (2%), Native American (1%), and Mixed (2%) with a mean age of 18.94 years (*SD* = 0.84, *Range* = 18–21).

### Design

The experiment used a mixed-subjects design with a manipulation of shared facial features (nose, eyes, mouth, control) and lineup race within subjects, and lineup type (simultaneous or sequential) between subjects. Lineups were randomly assigned to facial feature conditions across participants. All participants saw three shared-eyes lineups, three shared-nose lineups, three shared-mouth lineups, and three control feature lineups (i.e., lineups without a shared feature). Participants were randomly assigned to view simultaneous or sequential lineups. Each lineup included one target (i.e., all lineups were target-present), and lineup responses were forced-choice. Lineup positioning of the target was randomized in both lineup conditions; analyses were collapsed across lineup positioning. Lineup order was also randomized, as were the studied faces during the encoding phase.

### Apparatus

Participants’ eye movements were recorded using an SR Research EyeLink 1000 + tower mount eyetracker, sampling at 1000 Hz. Stimuli were displayed on a monitor 58 cm from the participants’ eyes, at a resolution of 1920x1080 px. The study was programmed in Python [36] using PsychoPy Coder [37].

### Materials

We used face stimuli from Dobolyi and Dodson [38]. Dobolyi and Dodson [38] developed these lineup materials from a face database developed by [39]. Twelve lineups were available in this stimulus set. All faces were male. The faces were presented in black and white to control for possible influences of color differences between photographs and were 416 x 540 px across all conditions: encoding, sequential lineups, and simultaneous lineups. These dimensions reflect the maximum possible face size that allowed all faces to fit on the screen in the simultaneous lineups. Only the head of the lineup members was shown.

#### Encoding.

Participants encoded six White target faces and six Black target faces. Each face was shown twice throughout encoding in random order with each face visible for three seconds per appearance. Two faces that were not used in the lineups were presented before and after encoding of target faces to mitigate primacy and recency effects.

#### Lineups.

**Simultaneous.** Participants viewed simultaneous lineups in a 2 x 3 array. Each target was presented alongside five fillers [38]. Lineups were adapted from Dobolyi & Dodson [38] under a CC BY license, with permission from Dr. Christian [39]. No person appeared in more than one lineup. Participants could select one of the six lineup members after a minimum of six seconds spent viewing the lineup.

**Sequential.** For the sequential lineups, participants viewed each lineup member one at a time, and each lineup was shown twice. Participants were presented with the sequential lineup in full before being allowed to make their identification decision. Specifically, participants were given the option of choosing of the six lineup members during the second run-through or after both run-throughs were completed. If a participant made their decision during the second run-through, the lineup stopped at that point.

#### Facial features.

We manipulated facial features (nose, eyes, mouth) using Adobe Photoshop. In all shared-feature lineups, we edited one feature on all of the lineup faces to match the target-face feature (either nose, or eyes, or mouth). To control for potential effects of editing itself, we edited the target’s feature back onto their face (imperfectly; i.e., slight changes in size and/or angle), and for the control condition, we edited one of each lineup member’s own feature back onto their face. That is, in the control lineups, each lineup member always had one of their own features edited (nose, or eyes, or mouth). The materials are available from the first author upon reasonable request.

### Procedure

After viewing the information sheet and providing informed consent, participants were instructed that they would be shown a series of faces (“We will now show you a series of faces. Please pay close attention to each face for the entire time that it is shown.”). Participants were not told that their memory for the faces would be tested. Participants were then presented with the encoding phase, which consisted of a two-face primacy buffer, followed by 12 target faces, followed by the 12 target faces again (randomly re-ordered), followed by a two-face recency buffer. After encoding, participants were informed that they would be asked to look at a series of lineups and make a lineup decision for each, and indicate how confident they were in that decision. In the simultaneous lineup condition, participants were instructed as follows:

We are now going to show you lineups to see if you can recognize the people that you just saw. Only one face per lineup will be the same as a face that was shown earlier. Each lineup will have 6 faces, with each face labeled with a number 1–6. To select the face you remember, press the corresponding key on the keyboard. The lineup will be shown for 18 seconds. You will have to look at them for at least 6 seconds before you can respond. You will also be given the opportunity to respond afterwards.

If participants ran out of time before making their selection, they were prompted with:

Which was the person you saw earlier? Use the numbers 1–6 on your keyboard to select the corresponding face.

In the sequential lineup condition, participants were instructed as follows:

You will see each face one at a time, each for 3 seconds. Each lineup will be shown twice.

After they are all presented, you will be asked to pick which one you remember seeing earlier. You can also pick during the second presentation of each lineup if you would prefer.

After the first run-through of each sequential lineup, participants were presented with the following instructions:

You will now see the same lineup one more time. After the lineup, you will be asked to pick the person you remember seeing at the beginning of the experiment. You can also make your response during the lineup if you would prefer.

After the second run-through, if participants had not already made their selection, participants were prompted with:

Which is the person you saw earlier? Use the numbers 1–6 on your keyboard to select the corresponding face.

Participants viewed either twelve simultaneous lineups or twelve sequential lineups. All lineups contained one target, and all studied targets appeared in a lineup. After each lineup decision, participants provided confidence judgments numerically (1–5, with anchors *not at all confident* to *completely confident*). Analyses of confidence can be found in the Supporting Information (see Figure S1 and Figure S2 in S1 File).

After the lineup task, participants completed a standard five-minute computerized visual reaction time task (CVRT). This allowed us to control for participants’ overall effort and attention to the experiment by using CVRT performance (standard deviation of reaction time) as a covariate in analyses [40]; Including CRVT as a covariate did not change our results; the open-access codes are available on the Open Science Framework). In the reaction time task, participants were repeatedly shown a single black X in the middle of the screen at varying ITIs (ranging from 250 ms to 2800 ms), and were asked to press the spacebar as quickly as possible whenever the X appeared. Finally, participants completed a demographics questionnaire along with questions about perception of the face stimuli (“Did you notice anything about the faces you saw? Feel free to describe anything, using all the space you need. There are no wrong answers.”), decision processes (“Did you use any strategy while looking at the faces?”; “What facial features did you look at first or focus on while making your decision?”), and task comprehension (“Did you have any suggestions for how the task could be improved or made clearer?”). We report responses to these measures in the Supporting Information Table S1 in S1 File. Participants were then presented with debriefing information and received course credit.

### Data reduction and analysis

#### Lineups.

Lineup decisions were coded as correct (target identifications) or incorrect (filler identifications).

#### Eye tracking.

We measured participants’ gaze in terms of (x, y) coordinates on the screen at 1000 Hz. We assessed the number of fixations and fixation duration to the eyes, nose, and mouth for each face. To control for potentially confounding effects of eye-tracking precision, we examined these measures as a proportion of total feature viewing; for example, we examined the proportion of fixations (or proportion of fixation duration) that were on the nose relative to other features. To define feature regions, an independent coder marked bounding boxes on the face regions for each feature of each face in Qualtrics (Provo, UT).

We measured the extent to which participants looked (i.e., number of fixations and fixation duration) at the edited feature in comparison to non-edited features in each lineup. For the shared feature conditions, this corresponded to the shared versus non-shared features, respectively. For the control condition, this corresponded simply to edited versus non-edited features, which allowed us to control for potential effects of attention to edited features (irrespective of whether they are shared). We examined attention to edited features both at a trial level and over time within a trial. For temporal analyses in the simultaneous condition, we binned edited feature fixations by timepoint in a trial in terms of scaled time (% of time that elapsed in the trial). For the sequential condition, we examined edited feature viewing over the course of sequential faces in a lineup.

#### Statistical analysis.

Data analyses were conducted using linear (for continuous outcomes) or binomial logistic regression (for binary outcomes) mixed effects models. For all analyses, we included a random intercept of target and a random slope of participant in the regression models (e.g., accuracy ~ lineup type + (lineup type |participant) + (1|target), Barr et al., [41]). However, note that the conclusions were equivalent when subject-level analyses were conducted (i.e., averaged scores for each subject for each condition, with a random intercept of subject). For comparison of shared versus control conditions, the shared feature conditions were each coded as shared. Effect sizes are reported as a standardized regression coefficient (β) for continuous predictors or as Pearson’s *R* for binary predictors. For all analyses, we regarded false discovery rate corrected [42] *p* values less than.050 as statistically significant.

To quantify the evidence in favor of the null for the key nonsignificant result obtained using frequentist statistics, we computed Bayes factors (using the *BayesFactor* package lmBF function in R with 5000 iterations and the default prior, [43] and assessed BF_10_—that is, the ratio of the Bayes factor in favor of the alternative hypothesis to the Bayes factor in favor of the null hypothesis. By convention, a BF_10_ < 0.33 indicates substantial evidence for the null hypothesis, and a BF_10_ < 0.10 indicates strong evidence for the null [44]. For this analysis, linear mixed effects models were used because the *BayesFactor* package does not yet support logistic regression. Therefore, subject-level models (averaging by condition by subject) were used for the Bayes factor analyses, with a random intercept of subject. These analyses were conducted by computing the Bayes factor of the comparison between a model with the variable of interest to the same model without the variable of interest. Specifically, because the interaction between condition and viewing edited features was the most direct test of our hypothesis, the models were as follows: 1) accuracy ~ (viewing photoshopped features) + (condition: shared vs control) + (1|subject), and 2) accuracy ~ (viewing photoshopped features) x (condition: shared vs control) + (1|subject). Note that the random effects were specified using the *BayesFactor* specification rather than “(1|subject).”

For viewing patterns, we analyzed both fixation count and fixation durations. These measures are conceptually and empirically related, though we chose to include both because they capture partially distinct aspects of visual attention (and are frequently employed together, e.g., [45]. Fixation count reflects how often participants orient to a region of interest (i.e., attentional allocation frequency), whereas fixation duration reflects the amount of time spent processing that region once fixated (i.e., attentional engagement or depth of processing). Although correlated, these indices are not fully redundant: theoretically, a manipulation could influence how frequently participants sample a region without affecting total dwell time, or vice versa. Therefore, we report our findings in relation to both.

#### Noticing of photoshopped features.

We coded participants’ qualitative responses during the debriefing questionnaire for mentions of alterations to the faces. To do this, we defined a keyword search function with a set of keywords based on the qualitative responses participants provided. A string-matching approach was then applied to count any mention to keywords using *stringr* [46]. Keywords were coded for “photoshop,” “photoshopped,” “AI,” “artificial,” “edited,” “altered,” “alterations,” “alteration,” “uncanny,” “unnormal,” “abnormal,” “copy and pasted,” “changed,” “combined,” “distorted,” “manipulated,” “composite,” “composites,” “augmented,” “deformed,” “generated.” The first author then manually checked the qualitative responses for coding accuracy. Only 19% of participants mentioned editing when asked if they noticed anything about the faces (e.g., “They all looked pretty normal. Some faces in the lineup looked weird like they had been photoshopped”). Because the noticing of face alterations could have influenced participants’ viewing behavior, we report analyses including and excluding these cases.

## Results

### Overall memory effects

We first examined whether feature condition (shared vs. control) and lineup type predicted overall accuracy, as well as whether their interaction significantly influenced accuracy. To do so, we fit separate models including fixed effects for condition and lineup type, followed by a model that additionally included their interaction term. This allowed for direct interpretation of any main effects.

**Fig 1:**
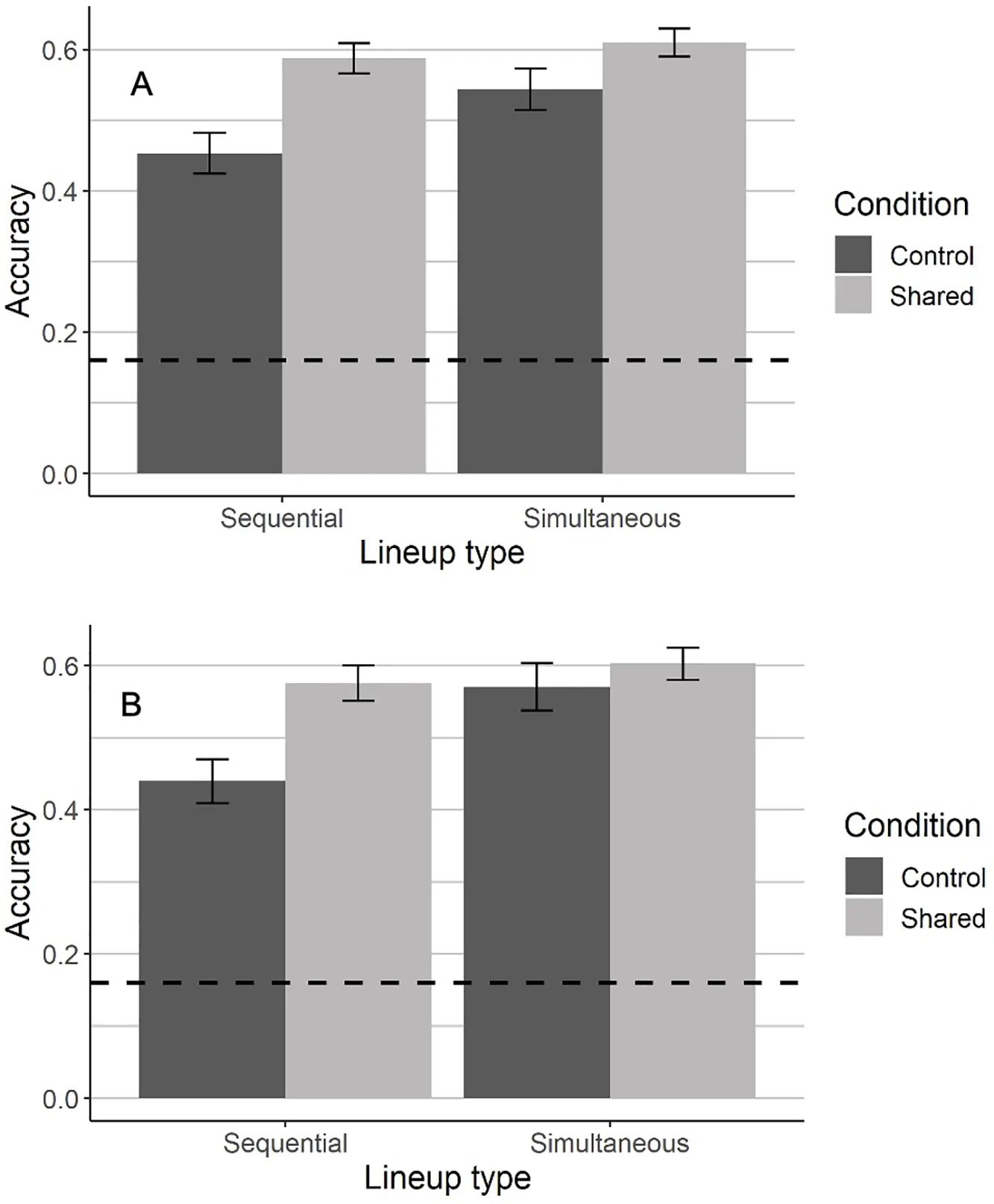
Accuracy by feature condition and lineup type. Note. Accuracy by feature condition A) including participants who noticed alterations, B) excluding participants who noticed alterations. The dashed line represents chance accuracy. Error bars represent the standard error of the mean.

Accuracy was significantly higher in the shared feature condition, suggesting that the presence of a shared feature *improved* performance, *B* = 0.61, *SE* = 0.20, *z* = 3.05, *p* = .005, r = .038. Lineup type did not influence accuracy, *B* = 0.15, *SE* = 0.20, *z* = 0.77, *p* = .443, r = .010. Condition and lineup type did not interact as predictors of accuracy, *B* = 0.51, *SE* = 0.40, *z* = 1.26, *p* = .277, r = .016. When participants who mentioned noticing alterations were excluded, accuracy remained significantly higher for the shared feature condition, *B* = 0.56, *SE* = 0.23, *z* = 2.49, *p* = .026, r = .034. Lineup type still was not a significant predictor of accuracy, *B* = 0.22, *SE* = 0.23, *z* = 0.99, *p* = .367, r = .014. The interaction between lineup type and feature condition was not a significant predictor of accuracy, *B* = 0.87, *SE* = 0.44, *z* = 1.98, *p* = .063, r = .028. In the shared feature condition, accuracy did not differ by lineup type (see Fig 1). In the control condition, accuracy was numerically higher for simultaneous lineups than sequential lineups.

### Relationship between viewing of edited features and accuracy

We then tested how viewing the edited features in each lineup, as a proportion of total feature viewing, predicted accuracy. We first tested this in terms of number of fixations and in terms of fixation durations as fixed effects, in separate regression models. Table S2 in S1 File reports full inferential results. Neither the number of fixations nor the duration of fixation to the edited features were significantly related to accuracy (.968 > *p*s > . 263,.019 > rs > .012).

We then tested whether there was an interaction between lineup type and viewing of edited features in predicting accuracy, by inserting lineup type as an interaction term with number or duration of fixations to the edited features (in separate models, e.g., accuracy ~ number of fixations to edited features*lineup type). We found no statistically significant interactions between lineup type and viewing of edited features (neither number nor duration) in predicting accuracy,.841 > *ps* > .463,.022 > rs > .005.

**Fig 2:**
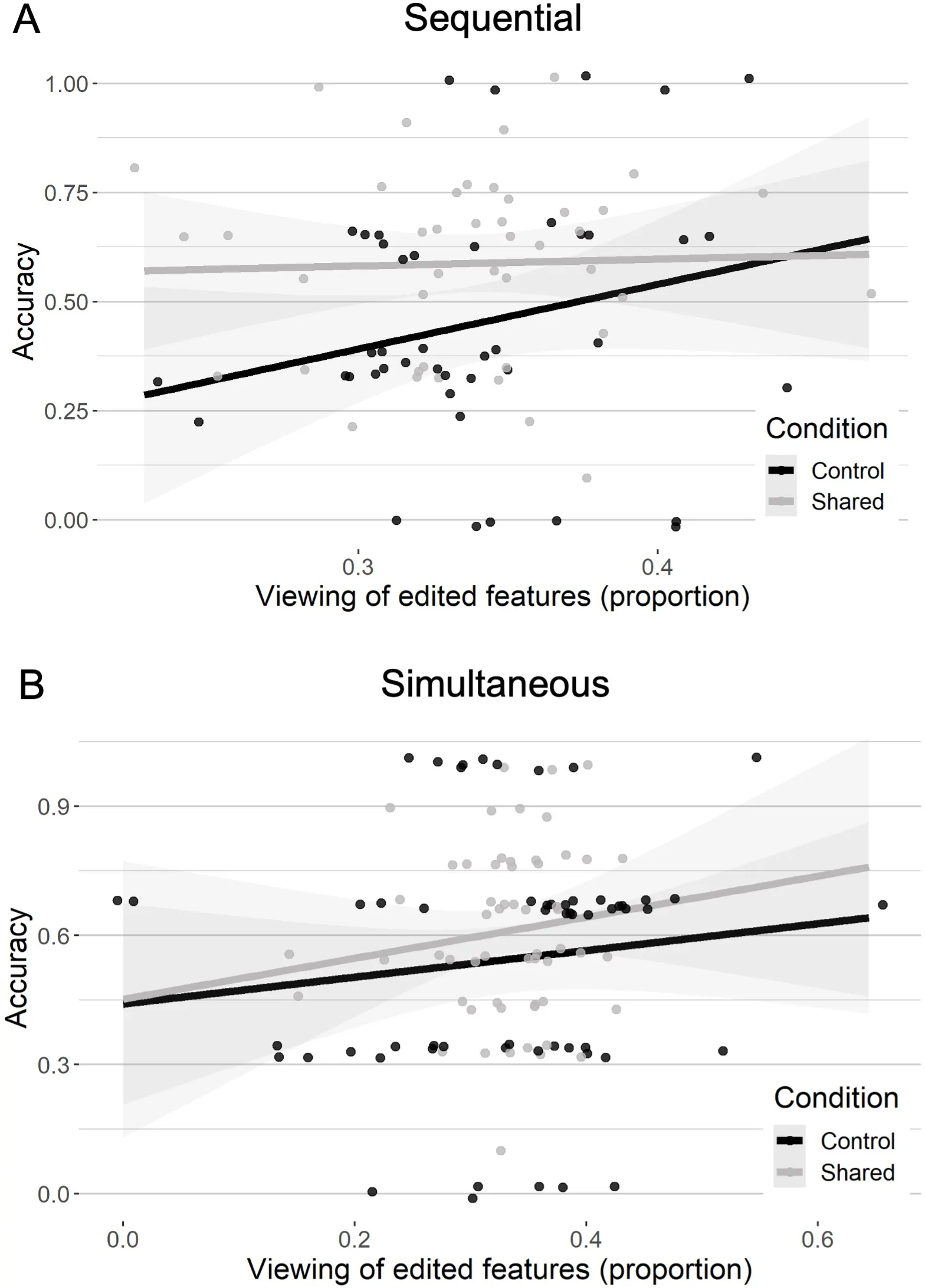
Accuracy by proportion of viewing of edited features in simultaneous and sequential lineups. Note. Lineup identification accuracy by proportion of fixation duration directed to edited features relative to other features in A) sequential lineups, and B) simultaneous lineups including those who noticed alterations. Any effects within the control condition reflect effects of image editing. Differences between conditions reflect effects specific to shared features. Points are jittered for visualization.

Importantly, however, these analyses do not control for potential effects of image editing itself. That is, it could be the case that viewing edited features in general predicts poorer accuracy—for example, reflecting distraction by image editing—but that viewing shared edited features predicts better accuracy than viewing non-shared edited features (i.e., in the control condition). Therefore, to provide the most direct test of shared feature viewing, we examined differences in effects between the control condition and shared feature conditions to determine whether there were any effects related to viewing shared features when potential effects of image editing are accounted for (i.e., when the control condition is used as a baseline). Specifically, we fit separate binomial logistic regression models predicting accuracy from either the number of fixations to edited features or the duration of fixations to edited features, along with condition (shared vs. control) as fixed effects, including their interaction term (e.g., accuracy ~ edited feature viewing × shared vs control condition). There was no significant interaction between shared versus control conditions and number of fixations, *B* = 0.08, *SE* = 0.23, *z* = 0.35, *p* = .949, r = . 004, nor between shared versus control conditions and duration of fixations, *B* = 0.02, *SE* = 0.31, *z* = 0.06, *p* = .956, r = .001, indicating that there was no relationship between viewing shared features and accuracy. Bayes factor analysis yielded substantial evidence in support of the null effect, BF_10_ = .24 (duration: BF_10_ = .26), suggesting that this lack of effect was not due to a lack of power. Fig 2 illustrates accuracy by proportion of viewing edited features (duration of fixations) by condition (shared vs. control) and lineup type.

We then ran all the models again, but this time excluding participants who mentioned noticing image editing. The pattern of findings was not different than those observed with the dataset including participants who mentioned image editing (see Supporting Information Table S3 in S1 File for full inferential results). There were no significant effects of viewing edited features on accuracy or interactions with lineup type or condition,.980 > *p*s > .540,.024 > *r*s > .001. Overall, therefore, these results indicate that viewing diagnostic features did not predict accuracy—suggesting that evaluations of diagnostic versus non-diagnostic features proposed by DFD may not involve overt attention.

#### Temporal analyses.

We next examined whether there may be specific temporal patterns of edited feature viewing that predict accuracy. For example, as outlined in the introduction, it could be that early viewing of shared features followed by a reduction in viewing of shared features over time predicts accuracy. Given that we used time-binning for this analysis in simultaneous lineups, we examined edited feature viewing in terms of the number of fixations. Specifically, for simultaneous lineups, we averaged the number of fixations made to edited features (as a proportion of total feature viewing) for each of 10 equal timepoints from lineup onset to lineup offset, such that each trial was split into time increments corresponding to 10% of total trial time. To ensure model convergence, scores were averaged by subject by condition within each time bin for these analyses, and linear mixed effects models with random effects of subject were used. However, results were equivalent when effects were examined at the trial level, with logistic mixed effects regressions when accuracy was the outcome. There were no significant changes in viewing edited features over time during the course of lineup viewing either linearly, β = −.02, *p* = .58, or quadratically, β = .05, *p* = .10. The interaction of time with viewing of edited features did not predict accuracy either for a linear effect of time, β = −.01, *p* = .46, nor a quadratic effect of time, β = −.02, *p* = .23. For the more direct test of shared feature viewing, there was also no interaction by feature condition linearly, β = .02, *p* = .58, or quadratically, β = −.03, *p* = .53. Bayes factor analysis indicated substantial evidence supporting the null hypotheses, BF_10s_ < .18. Results remained unchanged when participants who mentioned alterations were excluded, all *p*s > .09. This indicates that viewing of edited features did not consistently change over time during simultaneous lineups, and temporal changes in shared feature viewing did not predict accuracy.

For sequential lineups, we examined viewing of edited features within the first lineup presentation (after the first face, given that it would be impossible to notice that a feature is shared between lineup members when viewing the first face). There were no significant changes in viewing edited features over the course of face presentations linearly, β = .02, *p* = .63, or quadratically, β = .0004, *p* = .99. The interaction of time (i.e., face presentation number) with viewing of edited features did not predict accuracy either for a linear effect of time, β = −.03, *p* = .48, or a quadratic effect of time, β = −.03, *p* = .38. For the more direct test of shared feature viewing, there was also no interaction by feature condition linearly, β = −.01, *p* = .22, or quadratically, β = −.09, *p* = .31. Bayes factor analysis indicated strong evidence supporting the null hypotheses, BF_10s_ < .05. Results remained unchanged when participants who mentioned alterations were excluded, all *p*s > .20. This indicates that viewing of edited features did not consistently change over time in terms of the course of face presentations in sequential lineups, and temporal changes in shared feature viewing did not predict accuracy.

#### Self-reported decision strategy.

We coded participants’ qualitative responses regarding their decision strategy during the debriefing questionnaire for reference to features. When asked if they utilized any strategy while looking at the faces, 56% mentioned features (e.g., “I looked at hair, eyes, mouths”) while 44% did not report using a feature-focused strategy (e.g., “just whichever looked the most familiar).

### Follow-up analyses

#### Comparing simultaneous to sequential control lineups.

Because there has been controversy concerning the advantages of sequential lineups as opposed to simultaneous lineups (e.g., [16,47,48]), we compared performance between simultaneous and sequential lineups in our control condition. To do so, we fit a binomial logistic mixed effects model assessing the effect of lineup type (simultaneous or sequential) on accuracy for control lineups only. There was no significant difference between the simultaneous lineup and the sequential lineup, *B* = 0.69, SE = 0.48, *z* = 1.46, *p* = .287, *r* = .036. When partici*p*ants who noticed image editing were excluded, the pattern of results did not change, *B* = 1.12, SE = 0.53, *z* = 2.10, *p* = .071, *r* = .058.

#### Differences in viewing between conditions.

To further understand how the presence of shared features may influence decision-making processes during lineup viewing, we next examined whether the condition manipulations influenced feature viewing in terms of number of fixations and duration of fixations. There was no significant difference between the control condition and shared condition in viewing edited features in terms of number of fixations, *B* = 0.01, SE = 0.01, *z* = 0.60, *p* = .553, *r* = .008, or duration of fixations, *B* = 0.01, SE = 0.01, *z* = 0.42, *p* = .673, *r* = .005. This further suggests that the evaluation of diagnostic versus non-diagnostic features proposed by DFD may not involve shifts in overt attentional patterns. There was also no evidence of a significant interaction between feature condition and lineup type for both number of fixations, *B* = 0.01, SE = 0.03, *z* = 0.21, *p* = .850, *r* = .003, and duration of fixations, *B* = 0.01, SE = 0.03, *z* = 0.17, *p* = .863, *r* = .002.

Results remained unchanged when participants who noticed image editing were excluded. There was no significant difference between the control condition and shared condition in viewing edited features in terms of number of fixations, *B* = 0.01, SE = 0.01, *z* = 1.21, *p* = .227, *r* = .017, or duration of fixations, *B* = 0.01, SE = 0.01, *z* = 1.22, *p* = .220, *r* = .017. There was no significant interaction between feature condition and lineup type for both number of fixations, *B* = 0.02, SE = 0.03, *z* = 0.46, *p* = .690, *r* = .006, and duration of fixations, *B* = 0.02, SE = 0.04, *z* = 0.53, *p* = .596, *r* = .007.

#### Differences in viewing and accuracy by race.

There were no significant differences in identification accuracy for White compared to Black lineups, *B* = 0.28, SE = 0.43, *z* = 0.65, *p* = .518, *r* = .008. There was no significant interaction between race and feature condition, *B* = 0.03, SE = 1.16, *z* = 0.17, *p* = .866, *r* = .002, nor between race and lineup condition, *B* = 0.22, SE = 0.34, *z* = 0.64, *p* = .573, *r* = .008. Therefore, our findings described above, in which accuracy was higher for the shared feature than control condition, did not significantly differ by race.

Given known differences in facial feature viewing for faces of different races, we next conducted a secondary analysis examining feature viewing by race. To do so, we fit linear mixed effects models with proportion of facial feature viewing as the outcome (for example, the proportion of fixations made to the eyes, out of the total number of fixations made to facial features) and race (White or Black lineups) as the predictor. For all inferential results, see Supporting Information Table S4 in S1 File.

In White compared to Black lineups, participants viewed the eyes more, but viewed the mouth less (*p*s < .003, *r*s > .041), while viewing of the nose was not influenced by race (*p*s > .286, *r*s > .008). Race and lineup type did not interact in a statistically significant manner in their influence on viewing of eye and mouth (*p*s > .09, *r*s > .014), but they did in their influence on number of fixations to the nose (*p* = .007, *r* = .037). Participants viewing White faces were more likely to look at the nose if they completed sequential lineups rather than simultaneous lineups. Meanwhile, viewing of the nose was similar for Black faces across lineup types.

## Discussion

We tested eyewitnesses’ viewing behavior and decision-making when features amongst lineup members are shared (i.e., non-diagnostic) for simultaneous and sequential lineups. We expected that spending less time looking at shared facial features (i.e., fewer fixations, shorter duration of fixations) relative to other facial features would predict higher accuracy in identifying previously seen faces. We found mixed support for this hypothesis. In short, the presence of shared features improved accuracy, but there were no significant effects with respect to viewing behavior.

First, lineups with shared features (thus more similar) produced significantly higher accuracy than control lineups. Notably, our eye tracking results indicated that visual attention to shared features was not associated with accuracy, with substantial Bayesian evidence for the null effect. The overall increase in accuracy observed in the shared-feature condition may be driven primarily by participants who responded on the basis of a more global familiarity signal (e.g., [49]. Shared features may increase general familiarity across the lineup, potentially facilitating choice behavior. Alternatively, shared features may affect face processing via a feature-face congruency effect. As similarity amongst lineup members’ features increases, eyewitnesses may become more likely to infer the common source around which the lineup was constructed (i.e., in our case, the guilty suspect). As a result, the shared feature is perceived as most configurally coherent within the target face. The target may therefore “stand out” at a more implicit level (“Which face is the best example of the person I saw?”), leading observers to select them based on a sense of greater representativeness. In short, the target face may be selected because it offers greater holistic processing fluency (e.g., [32,50–52]), not because the shared feature is uniquely diagnostic.

Because shared features do not differentiate the guilty suspect from fillers, allocating greater attentional resources to them should not enhance accuracy. Indeed, while lineups with shared features produced significantly higher accuracy than control lineups, there were no differences in allocation of visual attention to the edited feature between conditions. In fact, for simultaneous lineups in particular, the numeric trend was in the opposite direction of what would be expected based on DFD: More viewing of edited features was (nonsignificantly) related to higher accuracy, particularly for the shared condition (see Fig 1). That is, more viewing of *non-diagnostic* features predicted nonsignificantly higher accuracy. In terms of edited feature viewing overall, the shared feature condition did not significantly differ in how long participants looked at the edited feature compared to the control condition in either lineup type. In short, allocation of overt attention to non-shared features did not predict differences in eyewitnesses’ accuracy, neither in simultaneous nor sequential lineups.

We expected that once eyewitnesses realized that features were shared among lineup members (i.e., after an initial assessment), they should spend less time looking at the shared features. We also did not find support for this hypothesis. There were no significant changes in shared feature viewing over time in sequential lineups nor simultaneous lineups. This finding does not support what we expected based on DFD, as it suggests that participants did not differentially allocate their attention based on the diagnostic value of features or, alternatively, failed to evaluate them as diagnostic versus non-diagnostic. However, in support of DFD, when participants who noticed alterations were excluded, accuracy was higher in simultaneous control lineups compared to sequential control lineups.

A potential explanation for our findings may be that participants adopted a visual search strategy for a shared feature in every lineup, potentially spending time looking for a shared feature while simultaneously deciding which face they had seen before. However, excluding participants that indicated that they noticed that the faces were altered did not change our results, suggesting that the effects were likely not driven by noticing the editing manipulation. Even if eyewitnesses did spend time looking for a shared feature, our findings indicate that they do not immediately realize which features are or are not diagnostic. Taken together, our findings suggest that DFD may not occur via allocation of visual attention to features, or that eyewitnesses do not overtly evaluate features as diagnostic.

There are at least two possibilities for why overt visual attention may not be involved in DFD-like processes. One possibility is that paying attention to diagnostic features only involves covert attentional shifting; that is, attentional shifts that do not involve eye movements, such as attending to one region while the eyes are directed at another. However, given that participants were able to freely move their eyes while evaluating lineups, there is not a clear reason why covert attentional shifts would be involved. More importantly, we found clear evidence that participants were indeed overtly examining facial features via eye movements, and that we had the power to detect effects on facial feature viewing; for example, there were substantial and highly significant differences in which features were viewed based on lineup type and lineup race.

A second possibility is that differences between diagnostic and non-diagnostic features may arise at the level of the memory matching process itself, separately from the evaluation and decision-making process. That is, assessments of global memory strength (i.e., memory strength not based on any individual feature) for the faces may become more sensitive when non-diagnostic features are present, perhaps by eliminating a source of non-mnemonic noise that could hinder performance. To that extent, overt attention to individual features may not be needed to determine the best match-to-memory. Since the shared feature originated from the target’s face, it may have been more seamlessly integrated into the overall concept of the target’s face compared to faces of filler lineup members (e.g., [53,54]), thus providing a better relative match-to-memory in the shared feature lineups compared to the average memory strength of lineup members. Future studies are needed to test these possibilities. The present results, however, suggest that the improvement in performance did not occur via changes in visual attention to shared features.

Our results suggest that overt visual attention may not be the mechanism by which diagnostic feature detection occurs. Mickes and Wixted [15] proposed that the simultaneous presentation of faces “allows the eyewitness to appreciate that certain facial features (i.e., those that are shared by everyone in the lineup) are non-diagnostic of guilt” [15]; p. 262). However, this proposition seems to imply that witnesses *pay attention* to said feature and *evaluate* it for its diagnostic value. Our findings suggest that this does not happen in a way that involves overt shifts of visual attention. Participants did not spend more or less time looking at the shared feature relative to unshared features.

Separately from the central question, we replicated previous work in that we found that participants viewed the eyes less and mouth more for Black compared to White faces (e.g., [55]). However, there were no differences in accuracy by lineup race. These findings are in line with previous work that used the same materials (but without the feature editing we did; [38]. Dobolyi and Dodson [38] manipulated whether participants saw same-race or different-race faces in simultaneous and sequential lineups. They report little effect of same-race and different-race face viewing on performance. Similarly, Grabman et al. [56] reports no differences in lineup accuracy between target-present Black lineups and target-present White lineups in their five-minute retention interval condition (i.e., the most similar condition compared to the present study). We used these materials because of their previously demonstrated suitability for repeated lineup designs; although race of lineup is manipulated in these materials, a cross-race effect was not a central question in the present study. However, our findings add to a growing body of work that feature viewing varies by face race [33,34,57].

The present research is among the first to use eye tracking to understand the mechanisms underlying lineup decision-making, but there are limitations that must be acknowledged. Materials used in the present research already suggest a high degree of global similarity as fillers were a priori matched to the target on broad characteristics (i.e., young male, White/Black; i.e., the lineups we used were already fair). Differences in overall similarity could affect allocation of overt attention. The degree to which diagnostic features are attended to may vary when global similarity in a lineup is lower than that of the stimuli we used—for example, when lineups are biased. In our shared feature lineups, the shared feature for fillers was edited to match the face of the target lineup member. It is possible that the degree of similarity of a specific feature affects processing as suggested by DFD. However, this does suggest that definitions are needed for *how similar* (or *different*) features need to be for them to be considered non-diagnostic (or diagnostic). Although most studies of DFD to date use identical features, eyewitnesses are unlikely to view identical features in real world lineups; therefore, the extent to which DFD can explain eyewitness decision-making in real world scenarios is not yet established. Though our data are limited in addressing this issue, we encourage future research to develop these definitions.

Another consideration is that holistic face processing leads people to evaluate features as part of an integrated face percept, rather than evaluating facial features separately (e.g., [58–61]). Much of the evidence in support of DFD involves distinctive features, such as specific tattoos, bruises, or scars (e.g., [16,17]). Presence of such features may affect how faces are viewed and recognized. Distinctive features differ from naturally occurring features because they, by definition, draw attention (e.g., [62]). There is also evidence to suggest that distinctive features are processed as objects, rather than an extension of the face (e.g., [63]). As such, eyewitnesses may allocate visual attention specifically to features when they are particularly distinctive or novel, potentially limiting the generalizability of DFD to features that are non-distinctive, such as the naturally occurring facial features used in the present study. Relatedly, memory strength may also influence the extent to which DFD processes may occur. When memory is strong, eyewitnesses may not evaluate faces relative to one another at all (e.g., [64]; [65]). Future research should test whether features are attended to differently under varying memory strengths.

As the first step in probing the role of overt attentional mechanisms in the effects of diagnostic versus non-diagnostic features, we utilized target-present lineups only in a forced-choice paradigm. Importantly, we always edited the *guilty* suspect’s feature to be shared amongst lineup members in the shared feature lineups. We thus cannot draw inferences about decision-making on lineups when the suspect is innocent. This is an important question for follow-up studies to address. In real-world police procedures, lineups may be target-absent, and witnesses retain the option to reject the lineup. The absence of target-absent trials in the present study means that we could not assess whether shared features would influence innocent-suspect identifications. It may be that a salient shared feature across lineup members in a target-absent lineup increases overall choosing rates and thereby false positives. Accordingly, the present findings only speak to one side of the diagnosticity equation (i.e., how shared features affect the accurate identification of guilty suspects). Future work may address whether shared features improve discriminability between guilty and innocent suspects.

Another potential limitation concerns the ability of the present design to detect subtle effects. Compared to eye tracking studies in the broader memory and face processing literatures (e.g., [66–68]), our trial numbers were relatively low (twelve lineups per participant). However, given the repeated lineup design, our trial numbers were high compared to typical eyewitness paradigms (e.g., [69–71]). In a similar vein, because the use of simultaneous lineups requires that many faces be presented at once, the stimuli in the present study are quite small and thus could produce lower-precision eye movement measures than standard single-stimuli eye tracking studies. Therefore, it is possible that we were simply unable to detect differences in visual attention to shared features. However, we observed highly significant differences in viewing of specific facial features between lineup types and lineup race (including small effect sizes and interactions), which were replications of previous effects (i.e., highly unlikely to be spurious). This suggests that we did have sufficient power and eye tracking precision to detect small-to-moderate effects. Moreover, analysis using Bayes factors revealed substantial evidence in favor of the null hypotheses. Together, this suggests that the lack of relationship between shared feature viewing and accuracy in our paradigm is likely robust. A potential direction for future work would be to examine temporal dynamics in greater detail, such as whether the first fixation, the final fixation prior to identification, or specific transition sequences between facial features (e.g., eyes, then mouth, then eyes) predict accuracy. Although such analyses were beyond the scope of the present preregistered research questions, they may provide deeper insight into the mechanisms underlying diagnostic feature detection.

## Conclusion

We tested eyewitnesses’ decision making on simultaneous and sequential lineups using eye tracking. There were no differences in allocation of overt visual attention (via eye movements) to diagnostic features between lineup types or by accuracy, though shared-feature lineups resulted in higher accuracy compared to non-shared feature lineups. Our work suggests that diagnostic feature detection may not occur via allocation of visual attention to facial features, or that eyewitnesses do not overtly evaluate facial features as diagnostic. Our findings suggest that mechanisms besides overt attention are needed to explain how eyewitnesses may assess the diagnostic value of features in lineups.

## Supporting information

S1 File Supporting materials. Supplementary figures and tables.
(DOCX)
