# Supplementary material for: Does visual attention to diagnostic facial features predict eyewitness identification accuracy? Implications for diagnostic feature detection theory
Source: PLoS One. 2026 Sep 22;21(9):e0355167. doi: 10.1371/journal.pone.0355167 (PMC13596790; doi:10.1371/journal.pone.0355167)
Supplement: Supplementary file 1 [file pone.0355167.s001.docx]

Supplemental Materials

**Table S1**

*Responses to Self-report Measures*

| Face perception | Decision strategy | Focus on feature |
| --- | --- | --- |
| They were all relatively young | Noticeable features such as facial hair | Eyes and mouth |
| Sometimes the pictures look like the same face but different features are expressed either wider or in a different size. | First I looked at the persons eyes and then looked at their mouth/nose. | Eyes but then nose |
| The faces apparead to be photoshopped | I looked for evidence of photoshopping | Eyes and nose |
| They all seemed to have some sort of deformity, abnormal feature, and they were all male. | I tried to find a feature to remember. | symmetry of the ears, nose, overall face shape, eye width, and I think that is all. |
| it looked like there was one feature copy and pasted on each face in some of the line ups for example there was many that had the same nose shape | no | eyes and smile |
| Mostly face shape, distance of the features i.e. eyes, eyebrows, nose to mouth | No | Face shape |
| They all looked pretty familiar. | No | Nose and ears |
| Not really other then that every single personas eyes were shaped differently from each other. | I try to remember eyes and hair when looking at peoples faces. | The eyes |
| some faces were really familiar and some I did not remember at all | I tried to remember the hair style and different features they had | how each nose was or the different hair style the men had |
| the ones before were smilning and the ones in the one ups were not | no | smile |
| Towards the end, some of the same faces were shown | No | Nose and eyes |
| after each round all of the faces started look the same and I had a harder time deciding | A lot of them had shiny noses so I would typically pick that person | their nose |
| some guy had really pointy ears. Some of them had similar features. | I mostly looked at their mouths and ears. | mouths, ears, and chins |
| some of them looked distorted on the noses and lips | nope | nose |
| I feel like I have seen maybe a few from other various studies I have done, but not many. | tried to look for unique things about their facial or body features (if I could see parts of their body) | I think their eyes |
| I noticed that some of them looked like they were edited or deformed in the lineup | I remembered faces from the beginning more, as it got towards the end I could not remember as many faces | nose, hair, head shape |
| I noticed most had similar facial expressions and there seemed to be a pattern with the way the faces were shown | it seemed to be one smiling the next face was not. Also it seemed to be a white person then a black person. | there eyes, hair and mouth |
| Several of the faces appeared to be photoshopped. | I looked at their noses a lot. | I looked at the nose and the eyes. |
| I tried to focus on the eyes the most or an important feature. | Looking at the eyes | Eyes |
| In each lineup, the faces were all similar but seemed kind of distorted in different ways. | No | nose |
| they were all adult males | no | hair, smile, ears |
| A LOT OF THEM HAD BUZZED HEADS. ONE GUY HAD AN ADAMS APPLE/ ONE GUYS NECK LOOKED LIKE HE WAS SUCKING IN. | IT IS NOT REALLY A STRATEGY BUT I NOTICED I TEND TO LOOK AT THEIR MOIUTHS AFTER THEIR EYES AND THEN THEIR FOREHEAD AND HEAIR LAST. | THEIR EYES AND MOUTH UNLESSS THEY HAD SOME VERY DISTINCTIVE FEATURE THAT CAN HELP ME TELL THEM APART. |
| First thing I noticed on the faces were the eyes, and I would look at the mouth and hair. alot of them seemed to have similar hair and it was hard to tell that apart. sometimes the mouth would have facial hair and I would notice that stand out | I didn't really use any strategy I just looked the eyes. | I looked at the yes mostly and the mouth. |
| They were pretty forgettable faces. | tried remembering what there eyes looked liked | eyes |
| I feel like several of them shared similar characteristics even if the familiar one didn't. For example, I think I saw one with some sort of eyebrow slit. But given the array of faces many of them also had eyebrow "slits" but I think the one that I had seen previously didn't have one. | I was looking at facial hair and if they had any freckles/moles | I'm not really sure, I think I looked at eyebrows a lot for some reason. |
| Some looked familiar in facial structure, not only in race | No | Position of the eyes, shape of the nose, hair line |
| The faces appeared to have semi distorted noses often and pronounced mouths/ears. Certain faces were very distinct from the rest. | I focused on the unique feature, typically the nose. | The nose and then the lips or another obvious feature such as an afro |
| Some of them looked "uncanny" or like they weren't real. Also I felt during some of the trials I couldn't identify a face that looked anything like what was in the original lineup. | I associated some of the faces with faces I already knew | eyes, hair, nose, jaw structure |
| They all seemed like the same face but altered to look different. | I trusted by intuition. | eyes and nose |
| They were all similar according to hair and skin colour. | Not really | There face structure and neck/posture |
| Races of faces were never intertwined (i.e. white faces were only shown with other white faces). | Mainly looking at Jawline, ear, and hair shape | Jawline or ears |
| some were altered | I looked at their eyes | eyes and eyebrows |
| all were men probably in their 20's | find something unique about them, or give them a name | eyes, chin |
| I felt that some of the time I was shown a picture of the person but from a different angle and it was hard to recognize during those times. | I tried to see if they had any marks, moles, cuts, or areas where their facial hair grows. | I usually look at the persons nose and facial structure, but mostly distinguishing features like facial hair. |
| I felt like in 6 6 faces that were shown at a time they were all very similar to one another which made it difficult | not particularly I did look at one guys neck though which helped me | eyes and then hair then I would look fully at them |
| they were looking straight at the camera | no | eyes |
| It was hard for me to recognize most of them because they had the same filter, and they kinda look alike. | No | Lips |
| I noticed that they all didn't have their head faced the same way and some smiled while others didn't | I looked at their eyes and mouth to see with ones I recgonized | I looked at their eyes first and then their mouth/smile |
| it was a mix of races, face shapes, and overall faces structure. they looked similar when comparing | trying to look at the most prominent features | eyes, nose, chin, hairline |
| Some of the faces when it showed 6 next to each other looked distorted. | Not really | Eyes |
| nothing much, they were all in black and white picture, it was hard to determine certain facial features in a short time constraint | I tried to look more at the top half of the face | I tried to focus on the eyes and forehead and hair to match faces |
| The faces were all similar, it was hard to tell the difference when put next to other similar faces. | finding features that looked different on the person verses the others. | eyes and ears |
| Most of them looked like real people but some of them looked generated. They also didn't have many unique features that I could have remembered so I could be sure that I could select the right one. | I tried to remember their eyes and noses. | I think I looked at their nose first. |
| Some of the faces looked like the features didn't match or they were uneven, it made me think of the uncanny valley. | I tried to say the most normal looking faces were the ones I remembered but honestly they only seemed more familiar because they had normal features | The eyes |
| They were all different and none of them similar. I think all were positioned differently from one another whether it be tilt in the picture, different skin color, faces larger then others or different facial expressions. | I tried my best to remember certain features about such as, position of the face...etc | position of the face, charistics such as nose and eyes and also skin color. |
| In the line up some of the faces didn't seem recognizable at all. | Eye structure nose and ears | moles acne impurities in the face |
|  | no | eyes |
| I think most of them had large eyes | No | Eyes |
| The faces seemed like combinations of each other after awhile | I focused on the eye and nose area | I focused on the eyes, specifically the eye spacing |
| Most were African American and all males. | detached/attatches earlobes, hairline, lips, nose shape | lips |
| some had distinctive faces but for the most part they all had similar traits | I looked at hair, eyes, mouths | hair, eyes, mouth |
| After a while, I started to not recognize them. The ones in the lineup weren't smiling, and a lot of the original faces were smiling. | I just used my memory | I focused on head shape and mouth shape. I also looked at eyes a little |
| Some were somewhat looking the same while others were more obvious. | Yes, looking for unique facial features | Any scars or moles, and their eyes |
| They all looked alike and I had a hard time picking which one I saw previously | gut feeling | noses |
| No | No | nose |
| Yes, some of them had weird head shapes with pointy ears | Looked out for larger ears | lips |
| No I didn't notice any of the faces | remember certain features such as eyes, ears, etc | eyes and face structure |
| there was a couple faces I thought were repeated and those were the faces I was more confident about | look at eyes first | eyes |
| Some of the faces had more recognizable features, which helped me recognize them quicker during the actual experiment. I had less confidence in my answers when all 6 of the individuals shown had very similar characteristics. | I tried to look at each one for at least 3 to 4 seconds. | The eyes or the nose. |
| Some of the faces in the lineup looked more similar to the others in the lineup than others did. | no | eyes, nose, and mouth |
| it was reptitive with some of the faces | I looked at eyes | eyes |
| All semi relative features. | Jewelry, nose, clothes | Nose, chin, hair |
| I noticed that there was a fixture of ethnic people | looking at their hair | eyes |
| Most of the lineups all had similar facial structures | Find a unique thing in each face, so it would standout | Nose |
| Some of them looked distorted | Looked for one that looked normal. | Normality |
| it felt like I didn't recognize any. | Look at nose and mouth | Nose |
| Not really, they for the most part didn't have too many defining features that helped me pick them out among the other choices. | No | I have no clue. |
| They were all in black and white | focus on their nose and eyes | nose/eyes |
| most had straight faces | no | eyes or hair |
| some were very easy to spot, while others were harder. Some looked very very similar, which made me second guess whether my answer was correct or not/ | I would think about people they reminded me of, which made it a tad bit easier when choosing them. | face shape and eyes and mouth. |
| they all looked like mug shots | face structure | eyes I think |
| Most of the tip of the noses on the faces lined up with the dot. The faces were all males. | I just tried to really observe the eye shapes and their face shape. | eyes, nose, lips |
| They all looked similar | Tried to remember identifying features/facial imperfections | eyes |
| There were all different types of faces. All different races and all of the faces were men on the screen. Some of the faces looked very similar. | I would remember the types of noses and lips the faces had. | The nose or the lips. |
| I feel like the faces picked in the line up all had similar qualities to make it a little more difficult. | I tried to remember specific details about the picture or the person to try and be able to match them to that feature when looking at the lineup | eyes, nose, hair, and ears |
| they were inverted | just memory | eyes and eyebrows |
| There was so many faces that it was hard to determine if I had seen them in the past. | no | noses and hair. |
| The eyebrows and ears of the participants | facial features (ears, eyebrows, teeth) | eyes |
| Some looked distorted | The shape of their eyes | Eyes or nose |
| They all started to look unfamiliar towards the end. | I tried to focus on their eyes | I tried to look at their eyes and then the face as a whole |
| The people on the lineups looked similar | looking at facial features that stood out | Nose or eyes |
| I thought I recognized some facial features, but other features seemed different. so then I wasn't actually sure if it was a face I remembered seeing. | If there was a facial feature that stuck out to me, that is what I used to try to remember it. For example, If one eye seemed more open than the other, that is what I based my perception off of. | I mostly focused on the eyes, mouth, and ears. |
| They all looked pretty normal. Some faces in the line up looked weird like they had been photoshopped. | I tried to pick out distinctive features | Usually the noses, but sometimes the mouths were more distinctive. |
| not particularly | paid attention to who was smiling and other distinctive factors like facial hair and face structure. | eyes, mouth, and facial hair and teeth. |
| There was all male faces of either white or black | Not really no | eyes and nose |
| I remembered a few that had unique things like crooked eyes or a certain facial expression and I think that was the only ting that helped me choose any correctly. | Payed attention to unique facial features or expressions. | Eyes |
| no, the faces seemed completely ordinary. | I tried to remember certain features. | noses, eyes |
| I felt like the faces were not the same as the original. Some looked like their eyes or some feature had been changed. | just whichever looked the most familiar. | eyes, lips, hair, collarbones |
| I realized that some faces weren't on there, but they were all very similar in the way they all looked. There were also put together by color, making the line ups every other (white then black). | I used process of elimination when I saw a face I didn't recognize. | Their eyes and nose. |
| I did not notice anything specific about the faces | no | eyes |
| I didn't notice anything unusual about the faces while looking at them. I think they were all smiling in the first part, though. | I looked at certain facial features, especially noses and eye width. | Their nose and eye width/eye shape |
| Some of their eyes were bigger than others/made distinctive faces that made it easier to tell them apart. I also remembered one of the hairstyles one of the men had because it was different than all of the rest. | Yes | Eyes, lips, and hair |
| They seemed like they were combined | Tried my best to notice facial features | noses and facial hair |
| sometimes I would see a face in the test I thought id never seen before | id try and look at their haircut or forehead | haircut or eyes |
| I would notice the ears on a lot of the faces. I also noticed the chin and their hair a lot too. | Not really, I just tried to actually look at the faces and remember them. | The eyes and the ears. |
| Not really, but it was difficult to remember | No | eyes |
| Some of the faces shown felt like they weren't shown me to at the beginning | Not really | Eyes, hair, nose, neck |
| There were no women, so I only saw men. | No | Eyes and mouth |
| Most of them looked similar to each other | tried to remember distinct features | Nose, eyes, hair |
| I noticed on some of the sets there was similar face features and shapes but smaller portions were different about each. One of them specifically the faces all had similar structure, but they all had distinct nose flares so it was really just being able to tell them apart by minute features. | I tried to notice small hair differences and clothing but that didn't seem to help a ton with the smaller features. | Mainly hair, facial hair, sometimes eyes, and clothing |
| it felt like I didn't recognize any. | Look at nose and mouth | Nose |

*Note.* Responses to items A) face perception (“Did you notice anything about the faces you saw? Feel free to describe anything, using all the space you need. There are no wrong answers.”), B) decision strategy (“Did you use any strategy while looking at the faces?”, and C) focus on facial feature (“What facial features did you look at first or focus on while making your decision?”).

**Table S2**

Models With Viewing Patterns Predicting Accuracy Across Different Datasets

| **Model** | ***B*** | ***SE*** | ***z*** | ***p*** | ***r*** |
| --- | --- | --- | --- | --- | --- |
| Full Sample Dataset | | | | | |
| Number of fixations on edited features | 0.20 | 0.18 | 1.15 | .252 | .015 |
| Duration of fixations on edited features | 0.20 | 0.17 | 1.18 | .239 | .015 |
| Number of fixations on edited features*Lineup Type | 0.39 | 0.41 | 0.95 | .459 | .012 |
| Duration of fixations on edited features*Lineup Type | 0.30 | 0.38 | 0.80 | .496 | .010 |
| Dataset constrained to “Shared” features condition | | | | | |
| Number of fixations on edited features | 0.28 | 0.25 | 1.12 | .263 | .016 |
| Duration of fixations on edited features | 0.23 | 0.24 | 0.96 | .336 | .014 |
| Number of fixations on edited features*Lineup Type | 0.22 | 0.52 | 0.43 | .722 | .006 |
| Duration of fixations on edited features*Lineup Type | 0.15 | 0.45 | 0.32 | .841 | .005 |
| Dataset constrained to “Control” features condition | | | | | |
| Number of fixations on edited features | 0.10 | 0.22 | 0.47 | .968 | .012 |
| Duration of fixations on edited features | 0.16 | 0.21 | 0.77 | .876 | .019 |
| Number of fixations on edited features*Lineup Type | 0.91 | 1.48 | 0.62 | .717 | .016 |
| Duration of fixations on edited features*Lineup Type | 1.23 | 1.42 | 0.86 | .720 | .022 |

**Table S3**

Models With Viewing Patterns Predicting Accuracy When Participants Recognizing Photoshop Are Excluded

| **Model** | ***B*** | ***SE*** | ***z*** | ***p*** | ***r*** |
| --- | --- | --- | --- | --- | --- |
| Full Dataset excluding participants recognizing photoshop | | | | | |
| Number of fixations on edited features | 0.09 | 0.20 | 0.43 | .670 | .006 |
| Duration of fixations on edited features | 0.09 | 0.19 | 0.47 | .635 | .007 |
| Number of fixations on edited features*Lineup Type | 0.13 | 0.47 | 0.27 | .790 | .004 |
| Duration of fixations on edited features*Lineup Type | 0.01 | 0.46 | 0.01 | .997 | .001 |
| Number of fixations on edited features*Condition | 0.11 | 0.37 | 0.30 | .956 | .004 |
| Duration of fixations on edited features*Condition | 0.21 | 0.35 | 0.60 | .734 | .008 |
| Dataset constrained to “Shared” features condition | | | | | |
| Number of fixations on edited features | 0.17 | 0.28 | 0.61 | .540 | .010 |
| Duration of fixations on edited features | 0.12 | 0.27 | 0.44 | .658 | .007 |
| Number of fixations on edited features*Lineup Type | 0.08 | 0.59 | 0.14 | .976 | .002 |
| Duration of fixations on edited features*Lineup Type | 0.23 | 0.57 | 0.41 | .842 | .007 |
| Dataset constrained to “Control” features condition | | | | | |
| Number of fixations on edited features | 0.15 | 0.26 | 0.57 | .899 | .016 |
| Duration of fixations on edited features | 0.21 | 0.24 | 0.87 | .769 | .024 |
| Number of fixations on edited features*Lineup Type | 0.67 | 1.95 | 0.34 | .732 | .010 |
| Duration of fixations on edited features*Lineup Type | 0.93 | 1.65 | 0.56 | .576 | .016 |

**Table S4**

Viewing Patterns Across Race and Interaction of Race and Lineup Type

| **Model** | ***B*** | ***SE*** | ***z*** | ***p*** | ***r*** |
| --- | --- | --- | --- | --- | --- |
| Fixed Effect: Race | | | | | |
| Number of fixations on eyes | 0.04 | 0.01 | 3.70 | <.001 | .047 |
| Duration of fixations on eyes | 0.04 | 0.01 | 3.23 | .003 | .041 |
| Number of fixations on mouth | 0.05 | 0.01 | 4.55 | <.001 | .058 |
| Duration of fixations on mouth | 0.05 | 0.01 | 4.34 | <.001 | .055 |
| Number of fixations on nose | 0.01 | 0.01 | 1.09 | .286 | .014 |
| Duration of fixations on nose | 0.01 | 0.01 | 0.64 | .528 | .008 |
| Interaction: Race and Lineup Type | | | | | |
| Number of fixations on eyes | 0.04 | 0.02 | 1.63 | .105 | .021 |
| Duration of fixations on eyes | 0.03 | 0.03 | 1.09 | .275 | .014 |
| Number of fixations on mouth | 0.02 | 0.02 | 1.19 | .234 | .015 |
| Duration of fixations on mouth | 0.02 | 0.02 | 1.06 | .288 | .014 |
| Number of fixations on nose | 0.06 | 0.02 | 2.92 | .007 | .037 |
| Duration of fixations on nose | 0.05 | 0.02 | 2.16 | .063 | .027 |

***Confidence-weighted Accuracy***

As a follow-up analysis, we re-examined accuracy by weighting correctness by confidence judgments; scores for each trial ranged from -5 (maximum confidence incorrect response) to 5 (maximum confidence correct responses). Note that although we preregistered that we would examine confidence, we did not specify precisely how. These analyses used accuracy aggregated within groups. Figures S1 and S2 illustrate the confidence-weighted accuracy across conditions. The pattern of results was the same as the results obtained using the standard accuracy outcome: accuracy was higher in the shared feature condition, *SMD*=.31, *p*=.006, and all other effects were nonsignificant, *p*s>16.

**Figure S1**

*Confidence-Accuracy Relationship by Feature Condition*

**Figure S2**

*Confidence-Accuracy Relationship by Feature Condition and Lineup Type*
